# Aging in Place in Late Life: Theory, Methodology, and Intervention

**DOI:** 10.1155/2012/547562

**Published:** 2012-04-26

**Authors:** Agneta Malmgren Fänge, Frank Oswald, Lindy Clemson

**Affiliations:** ^1^Department of Health Sciences, Lund University, P.O. Box 157, S-221 00 Lund, Sweden; ^2^Department of Adult Education, Goethe University Frankfurt, 60325 Frankfurt-am-Main, Germany; ^3^The University of Sydney, Lidcombe, NSW 2141, Australia

This special issue focuses on aging in place in late life. Aging in place is about being able to continue living in one's own home or neighborhood and to adapt to changing needs and conditions. It is of high concern due to the increasing number of old and very old people in all societies and challenges researchers, practitioners, and policy makers in many societal and scientific areas and disciplines. We invited authors to contribute original research papers as well as conceptually driven review papers that would stimulate the continuing efforts to understand the different aspects of aging in place in late life. The papers that were submitted came from very diverse disciplines, such as sociology, psychology, occupational therapy, nursing, architecture, public planning, and social work. Given the number and diversity of papers submitted, we can conclude that aging in place is an important concern throughout the world and that different kinds of measures are taken to come up with local, national, and international solutions that enhance aging in place. It remains a very complex issue that needs and deserves to be investigated from many different perspectives and assessed by means of different methodological origin, covering qualitative and quantitative measures, as well as mixed-method approaches. Subsequently, the selection of papers presented in this issue only sheds light on some aspects of sociophysical person-environment exchange as people age, contributing to the ongoing discussion in the field of environmental gerontology.

Vasunilashorn et al. present a review study targeting the concept of aging in place as a research topic whose time has come. They found an increasing proportion of scientific papers over time, in particular those focusing on policy matters and the use of technology to support ageing in place. They concluded that aging in place is far from a one-size-fits-all issue but rather something that differs across populations due to, for example, culture, demographic, and legal systems.

The perspectives of the older persons themselves on social relationships and connectedness, social exclusion and inclusion, and the impact of the neighborhood were targeted in the following studies. By way of qualitative interviews, in the study by Emlet et al., older people were asked about their perception of social connectedness, how the society can help with life transitions to support aging in place, and what kinds of difficulties that they perceived in the home and neighborhood. However, different in conceptual framing and method, similar topics were emphasized by Yen et al., as well as Burns et al. The studies revealed that older people staying in the same neighborhood may experience strangeness, social exclusion, economic exclusion and insecurity due to gentrification and had few positive social ties in the neighborhood. They had a strong drive to stay active and to have meaningful social interactions with others, and they also wanted to contribute to the society. However, they experienced considerable structural barriers, for example, access to transportation services and other services in the neighborhood that made it difficult to stay active and connected to the society. Continuing on the same theme, a survey paper by Wu et al. investigated social isolation among older people in Singapore, finding that the strongest predictors were living alone or living with children. Also pointing towards the importance of community and social processes for aging in place the next paper by Galinsky et al. developed and tested a new measurement of collective efficacy feasible for use among older people. Collective efficacy refers to social processes on the level of person-neighborhood interactions, social cohesion, and informal social control, all known to be important for well-being in old age.

In contrast, indoor behavior may include various forms of person-environment relationships of more recent scientific interest. For instance, older adults with hoarding behaviors are often at risk of being evicted from their homes because they constitute a risk for other tenants' safety and security in the housing. For example, the risk of fire increases as does the sanitary risks of having a cluttered home. Thus, as Whitfield et al. pointed out in their paper, this group of people is at risk of being marginalized and to experience and rapidly declining health and well-being. The authors explored a collaborative community planning approach for finding solutions that could enhance the possibilities for aging in place. They found that, with structured collaboration between different actors in the communities, the professionals gained access to expertise from other staff and that such knowledge benefitted the community planning at large. The older people gained insight into their hoarding behavior, and they perceived that this approach fostered empowerment and minimized loneliness and isolation.

Yang and Sanford investigated the relationships between the environment, activity performance at home and community participation, and their potential for aging in place. Comparing older people with and without mobility limitations they found that persons with mobility limitations, experienced more environmental barriers in the home and the community than those without. They also found that environmental barriers in the home and the community explained travel and community participation among those with limited mobility. They reasoned that reducing environmental barriers in the home saves energy and the older person can thus be more active in the community.

The number of persons experiencing dementia “in place” is rising dramatically with increased age in the population. Their problems pose challenges to themselves but also to their close relatives and the society. Another study on aging in place with dementia by Beard et al. focused on couples where one partner had been diagnosed with dementia. In in-depth interviews, they expressed that they desired to go on as before and not to let the problems take over their lives. They strived to remain a couple and to invest as much energy as possible into a life where they worked together, developing a “joint career.” Investigating the management of dementia home care resources by way of an ethnographic design, Ward-Griffin et al. found that care resource allocation was relying heavily on family care giving and that formal resources were used as a supplement, most often when the family situation was becoming serious. Family care givers and recipients found the care system difficult to navigate in and without flexibility for acute needs.

One of many interventional approaches to support aging in place in late life is to offer preventive home visits to older people living in the community, mostly above a certain age. In some countries, it is mandatory for the municipalities to organize and conduct preventive home visits. The aim of the visit is to inform and identify current or potential risks to health, activity, and participation to be able to intervene before the problems occur. Different home visit protocols have been developed and applied in practice; however, the vast majority of them are not based on current evidence. In their study, Löfqvist et al. described the development and pilot testing of an evidence-based protocol for preventive home visits in Sweden. By way of reviewing scientific papers as well as conducting focus group interviews with older people, they identified key aspects important to include in the protocol. The protocol was then applied and tested for feasibility.

Finally, Jutkowitz et al. investigated post hoc the cost effectiveness of a home-based intervention targeting vulnerable older adults. The outcome was defined as life years saved. In the intervention group, the persons lived significantly longer, to additional costs for the intervention. Even though one can assume that the intervention group also may be healthier and consuming less health care resources, this remains to be investigated. To advance services and policies that support aging in place, economic analyses of programs are important. In this respect, the health economic approach used in the study offers a preliminary understanding of the costs of a highly effective intervention.

The variety in focus, theory, and methodology among the papers in this issue is a pleasing sign of the interest and effort being applied to aging in place issues by researchers and practitioners in diverse fields. Together and separately the papers have the potential to influence the societal debate as concern aging issues across the world and to inform decision makers in various fields about necessary measures to take in order to support aging in place in later life. We hope that the readers of this issue will find the papers interesting and inspiring for further research and debate.

*Agneta Malmgren Fänge* *Agneta Malmgren Fänge* *Frank Oswald* *Frank Oswald* *Lindy Clemson* *Lindy Clemson*

